# Genome-wide identification and expression profiling of the *carotenoid cleavage dioxygenase* (*CCD*) gene family in *Brassica napus* L

**DOI:** 10.1371/journal.pone.0238179

**Published:** 2020-09-03

**Authors:** Xin-Tong Zhou, Le-Dong Jia, Mou-Zheng Duan, Xue Chen, Cai-Lin Qiao, Jin-Qi Ma, Chao Zhang, Fu-Yu Jing, Sheng-Sen Zhang, Bo Yang, Li-Yuan Zhang, Jia-Na Li

**Affiliations:** 1 Chongqing Rapeseed Engineering Research Center, College of Agronomy and Biotechnology, Southwest University, Chongqing, China; 2 Academy of Agricultural Sciences, Southwest University, Chongqing, China; Huazhong University of Science and Technology, CHINA

## Abstract

Carotenoid cleavage dioxygenase (CCD), a key enzyme in carotenoid metabolism, cleaves carotenoids to form apo-carotenoids, which play a major role in plant growth and stress responses. *CCD* genes had not previously been systematically characterized in *Brassica napus* (rapeseed), an important oil crop worldwide. In this study, we identified 30 *BnCCD* genes and classified them into nine subgroups based on a phylogenetic analysis. We identified the chromosomal locations, gene structures, and *cis*-promoter elements of each of these genes and performed a selection pressure analysis to identify residues under selection. Furthermore, we determined the subcellular localization, physicochemical properties, and conserved protein motifs of the encoded proteins. All the CCD proteins contained a retinal pigment epithelial membrane protein (RPE65) domain. qRT-PCR analysis of expression of 20 representative *BnCCD* genes in 16 tissues of the *B*. *napus* cultivar Zhong Shuang 11 (‘ZS11’) revealed that members of the *BnCCD* gene family possess a broad range of expression patterns. This work lays the foundation for functional studies of the *BnCCD* gene family.

## Introduction

“Carotenoids” is the general term for the class of natural pigments widely present in animals, plants, and microorganisms. Carotenoids are lipid-soluble isoprene-like compounds that contain 40 carbon molecules and comprise more than 750 pigments with different structures [1]. These pigments have numerous important biological functions; for example, they are photo-protective and are indispensable components of photosynthesis [2, 3]. They also can scavenge free radicals and have antioxidant properties [4]. Furthermore, carotenoids are components of cell membranes, potential anti-cancer agents, and can interact with proteins [5]. Carotenoids are exploited as coloring agents in flowers and fruits to attract pollinators and agents of seed dispersal [6–8]. In addition, carotenoids are precursors of important phytohormones, such as abscisic acid and strigolactones, which regulate plant development and plant–environment interactions [9–11].

The carotenoid biosynthesis pathway in plants has been fully elucidated; the catalytic oxidative cracking of carotenoids is a key process in this pathway. Multiple conjugated double bonds exist within the carotenoid center chain, which can be specifically cleaved by carotenoid cleavage dioxygenases (CCDs) to form a variety of apo-carotenoids, and some apo-carotenoids can be further degraded into small biologically active molecules [12]. In plants, the addition of two oxygen atoms to the cleavage product means that CCDs possess the characteristics of a dioxygenase [13]. Moreover, CCDs are also a class of non-heme oxygenases, whose catalytic activity requires Fe^2+^ as a cofactor [14, 15]. CCD proteins contain four highly conserved histidine residues bound to Fe^2+^ and all contain a retinal pigment epithelial membrane protein (RPE65) domain that is characteristic of enzymes involved in carotenoid cleavage [16, 17].

In plants, CCD proteins are encoded by an ancient gene family. *CCD* gene family consists of two subfamilies: *carotenoid cleavage dioxygenases* (*CCDs*) and *9-cis epoxycarotenoid dioxygenases* (*NCEDs*) [12]. Through analysis of a novel ABA-deficient mutant of maize, the first protein found to specifically cleave carotenoids, viviparous14 (VP14), was identified by Schwartz *et al* [14]. Vallabhaneni *et al*. described the characteristics of the *CCD* gene family in the grass species maize (*Zea mays*), rice (*Oryza sativa*), and sorghum (*Sorghum bicolor*) [18]. During water stress and seed dormancy, *TaNCED* of *Triticum aestivum* might play a primary role in regulation of ABA content [19]. Besides, drought stress can induce expression of *NCED3* and accumulation of endogenous ABA in *Nicotiana tabacum* [20]. Wei *et al*. found at least seven *CCD* genes in *Solanum lycopersicum* genome sequence and analyzed their expression patterns [21]. *CCD* genes have also been identified or functionally expressed in a variety of other plant species, such as *Glycine max* [22], *Gossypium hirsutum*, *Solanum tuberosum* [23], *Cucurbita pepo* [24], *Saccharum officinarum* [25], *Crocus sativus*, *Osmanthus fragrans* [26], *Vitis vinifera* [27], *Mangifera indica* [28] and *Amygdalus persica* [29].

In *Arabidopsis thaliana*, the *CCD* gene family consists of nine members: four *CCD* genes (*AtCCD1*, *4*, *7*, and *8*) and five *NCED* genes (*AtNCED2*, *3*, *5*, *6* and *9*) [30]. Carotenoid cleavage dioxygenase homologs in other plant species are named according to the system used for Arabidopsis *CCD* family members.

The CCD1 and CCD4 enzymes catabolize a variety of carotenoids and produce volatile apo-carotenoids, which are important for the biosynthesis of aromas and flavors of flowers and fruits, respectively [31, 32]. The carotenoid content of mature seeds of *AtCCD1* mutants was higher than that in the wild type, indicating a role for *CCD1* in carotenoid catabolism [33]. *CCD4* regulates carotenoid homeostasis and *CmCCD4a* is specifically expressed in white petals of *Chrysanthemum morifolium* [34]. RNAi-mediated inhibition of *CmCCD4a* expression in white-petaled plants results in the production of yellow flowers [35]. In line with this observation, loss or downregulation of *CmCCD4a* function led to an increase in carotenoid content in petals [12]. *CCD7* and *CCD8* function in strigolactone biosynthesis, regulate axillary bud growth, and inhibit branching [36, 37]. Using RNAi to reduce *Actinidia chinensis CCD8* expression increases in branch development and delays leaf senescence [38].

The reaction catalyzed by 9-cis-epoxycarotenoid dioxygenase (NCED) is a rate-limiting step in ABA biosynthesis, and thus influences plant tolerance to diverse abiotic stresses [39]. *AtNCED5*, *AtNCED6* and *AtNCED9* are the dominant contributors to developmentally regulated ABA synthesis in seeds and thereby regulate seed embryo maturation and dormancy [30]. Overexpression of *OsNCED3* increased drought resistance in rice and caused an increased ABA level [40]. *AtNCED2* and *AtNCED3* transcripts are abundant in Arabidopsis roots and their encoded proteins function in abscisic acid biosynthesis and thereby regulate lateral root growth [30]. Furthermore, heterologous expression of *Brassica napus NCED3* led to ABA accumulation and NO and ROS generation in transgenic Arabidopsis plants, thereby enhancing abiotic stress tolerance [41].

*B*. *napus*, an important oilseed crop globally, is an allopolyploid derived from a natural interspecific cross between *Brassica rapa* (turnip; 2n = 2x = 20) and *Brassica oleracea* (kohlrabi; 2n = 2x = 18). The important physiological functions of carotenoid cleavage products in plants, such as abscisic acid and strigolactones, have prompted studies of the lyases involved in carotenoid metabolism. Little is known about the *BnCCD* gene family. The availability of the *B*. *napus* genome sequence would enable the identification and analysis of members of this family [42].

In this study, we identified 30 *BnCCD* genes. In addition to analyzing their gene evolution and structure, chromosomal localization, conserved motifs, and *cis*-acting promoter elements, we determined their tissue- and organ-specific expression and examined the physicochemical properties of their encoded proteins. The results form a solid basis for further studies on the biological functions of the *BnCCD* gene family.

## Materials and methods

### Plant materials

The *B*. *napus* cultivar Zhong Shuang 11 (‘ZS11’) was planted in Chongqing, China (29°45’ N, 106°22’ E). To analyze the expression patterns of *BnCCD* genes, four different tissues of ‘ZS11’ were harvested when plants were in full flower: mature leaves (Le), sepals (Se), flowers (F), and stems (St). The seeds (S) and silique pericarps (Sp) were harvested at different timepoints following the termination of flowering (25, 30, 35, 40, 45, and 50 days after flowering). Samples were immediately frozen in liquid nitrogen and stored at –80°C for further use.

### Identification of *CCD* genes in *Brassica*. *napus*, *Brassica*. *rapa*, and *Brassica*. *oleracea*

The coding sequences of *AtCCD genes* were downloaded from TAIR (https://www.arabidopsis.org/) and used as reference sequences. The *BnCCD*, *BrCCD*, and *BoCCD* genes were identified through the BLASTN analysis [43] of *AtCCD* genes against the Brassica Database (BRAD, http://brassicadb.org/brad/index.php) [44]. The local database was established using Geneious 4.8.5 software (http://www.geneious.com/; Biomatters, Auckland, New Zealand). The genome-wide alignment was verified by the MEGABLAST program [45], and the following screening standards were used: the consistency of aligned sequences with the reference sequence was ≥80% and gene sequences <700 bp were discarded. Because enzymes involved in carotenoid cleavage contain a retinal pigment epithelial membrane protein (RPE65) domain [16, 17], gene sequences from the Pfam database [46] that did not contain this domain were excluded.

### Multiple sequence alignment and phylogenetic analysis

CCD amino acid sequences were subjected to multiple sequence alignment using MUSCLE [47] with default parameters. The evolutionary relationships of *B*. *napus* CCD proteins with those of *A*. *thaliana*, *B*. *oleracea*, and *B*. *rapa* were analyzed using MEGA7 [45]. To identify the conserved blocks of all predicted sequences, the Gblocks program was used [48]. The substitution saturation was detected using DAMBE [49]. A phylogenetic tree was constructed using the neighbor-joining method implemented in MEGA7. The number of bootstrap replications was 1,000, and paired deletion was performed. The phylogenetic tree was visualized using FigTree v1.4.2 (http://tree.bio.ed.ac.uk/software/figtree/).

### Protein properties, sequence analysis, and duplication time inference

The chromosomal location of *BnCCD* genes, which was queried from the *Brassica napus* genome browser, and the genes were mapped onto chromosomal linkage groups by Mapchart software [50]. The Gene Structure Display Server (GSD 2.0) (http://gsds.cbi.pku.edu.cn/index.php) was used to portray the exon–intron structures of the *BnCCD* genes. The ExPASy proteomics server database (http://expasy.org/) [51]was used to predict the relative molecular weight, theoretical isoelectric point, protein stability, and aliphatic amino acid content of BnCCD proteins. The conserved motifs were identified using the MEME Version 5.0.5 online tool (http://meme-suite.org/tools/meme) [52] and the maximum motif retrieval value was set to 20; other parameters used the default settings. Annotations of the identified motifs were obtained from InterProScan (www.ebi.ac.uk/Tools/InterProScan/) [53].

To determine whether the CCD protein-coding sequences are under selective pressure, the ratios of synonymous substitution rate (*ks*) and non-synonymous substitution rate (*ka*) of homologous gene pairs were calculated using TBtools software [54]. The approximate date of duplication events was inferred by substituting Ks values into the formula $T=Ks/\left(2\times 1.5\times 10^{-8}\right)\times 10^{-6}$million years ago (MYA) [55].

### Predicted subcellular localization of BnCCD proteins and promoter analysis of *BnCCD* genes

The subcellular localization of BnCCD proteins were predicted using WoLF PSORT (http://www.genscript.com/tools/wolf-psort) [56]. The SOPMA secondary structure prediction method (https://npsa-prabi.ibcp.fr/cgi-bin/npsa_automat.pl?page=/NPSA/npsa_sopma.html) was used to predict the secondary structure of BnCCD proteins and the presence of transmembrane helices within BnCCD proteins were predicted using TMHMM Server v. 2.0 (http://www.cbs.dtu.dk/services/TMHMM/). The promoter sequences (2.0-kb region immediately upstream of the translation start sites) of the *BnCCD* genes were obtained from the *B*. *napus* genome database (http://www.genoscope.cns.fr/brassicanapus/) [57]. PlantCARE (**Error! Hyperlink reference not valid.**webtools/plantcare/html/) [58] was used to predict the presence of *cis*-acting sequences within each promoter.

### RNA-seq analysis

To analyze the tissue-specific expression of the *BnCCD* genes, publically available *B*.*napus* RNA-sequencing (RNA-seq) data PRJNA358784 (BioProject) were downloaded from the National Center for Biotechnology Information (NCBI, https://www.ncbi.nlm.nih.gov/) [59]. All *BnCCD genes* expression levels were quantified in terms of FPKM (fragments per kilobase of exon per million mapped fragments) using Cufflinks with default parameters [60], and then extracted from RNA-seq data according to their *B*. *napus* code, including expression data from thirteen different organs (roots, stems, leaves, buds, anthocauli, calyxes, petals, pistils, stamens, anthers, capillaments, seeds and silique pericarps) at different developmental stages in *B*.*napus* cultivar ZS11. The heatmap for *BnCCD* genes was constructed using TBtools software.

### RNA extraction and quantitative real-time PCR

Total RNA was extracted using the RNeasy Extraction Kit (Invitrogen, Carlsbad, CA, USA) according to the manufacturer’s instructions. Contaminating genomic DNA was removed with DNase I. Total RNA (1 μg) was used to synthesize cDNA by reverse transcriptase (TaKaRa). The gene-specific primer pairs used to analyze *BnCCD* genes expression by qRT-PCR were designed using Primer Premier 5 [61] and the *ACTIN 7* gene was used as an endogenous reference gene. All the primers were listed in S1 Table.

Quantitative RT-PCR was carried out using TB Green Premix Ex Taq on a Real-Time PCR Detection System (Bio-Rad, Hercules, CA, USA). The reaction mixture included 10 μL TB Green Premix Ex Taq, 0.5 μL forward primer, 0.5 μL reverse primer, 2 μL cDNA template, and 7 μL nuclease-free H_2_O in a total volume of 20 μL. The PCR cycling conditions were as follows: 95°C for 30s, followed by 40 cycles of 95°C for 5 s and 60°C for 30 s. Following PCR amplification, the dissolution curve was analyzed to ensure the specificity of the amplified products. Three biological replicates and three technical replicates were performed for each reaction. The relative gene expression levels were calculated using the 2^−ΔΔCt^ method [61]. All the results were plotted as the mean ± standard error of mean (SEM) from three independent biological replicates using Graph Pad Prism 5.0 software (GraphPad Software Inc., San Diego, CA, USA).

## Results

### Identification and characterization of *BnCCD* genes

**Fig 1:**
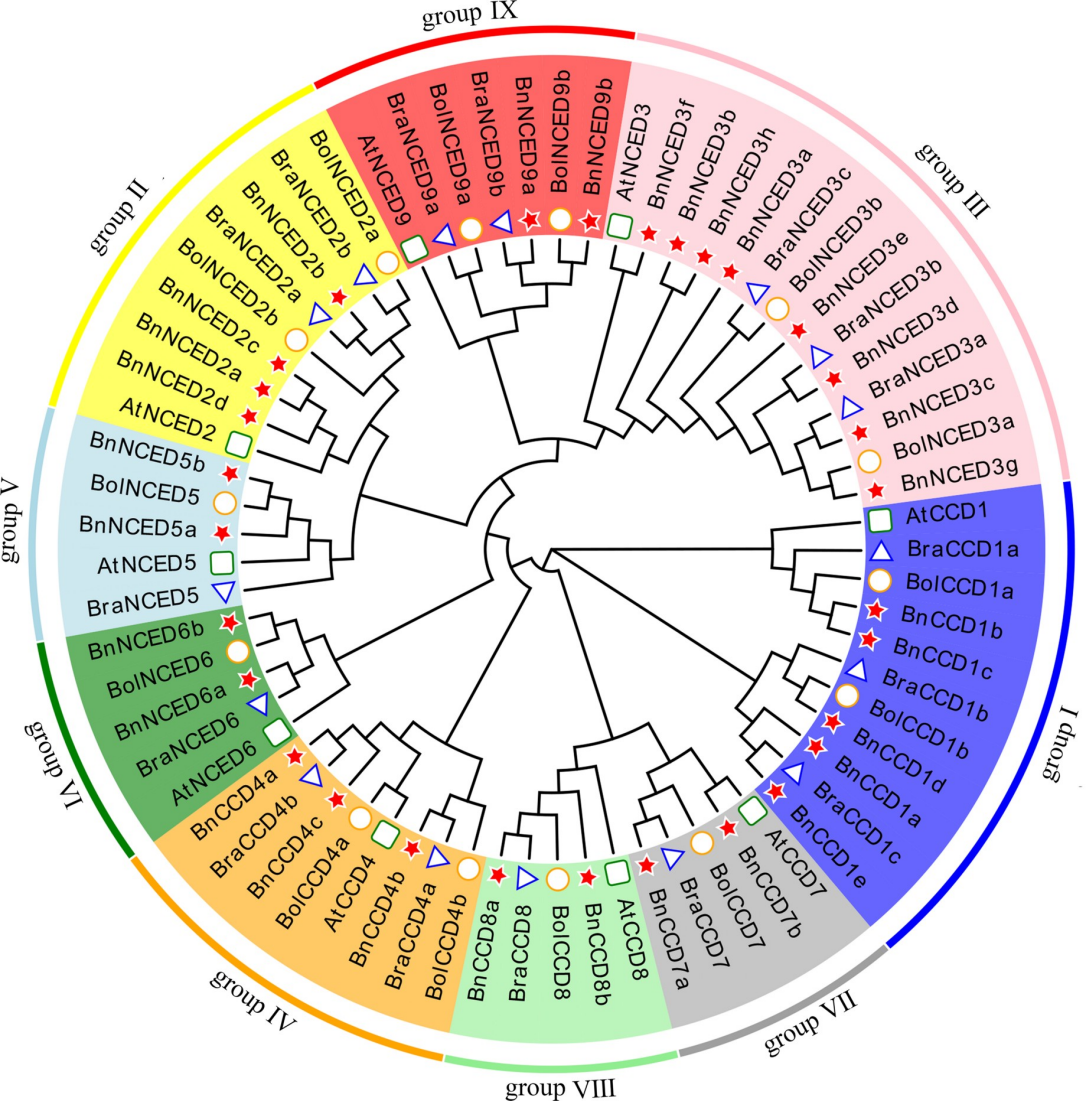
Phylogenetic analysis of 60 CCD protein sequences from *A*. *thaliana*, *B*. *rapa*, *B*. *oleracea*, and *B*. *napus*. Sixty CCD protein sequences were used to construct the neighbor-joining tree using Mega 7 with 1,000 bootstrap replicates. The CCD proteins were devided into nine phylogenetic subgroups (I, II, III, IV, V, VI, VII, VIII and IX) and are marked with different background colors.

Using the nine *AtCCD* coding sequences as a BLASTN query, we identified 30 *B*. *napus CCD genes* (*BnCCDs*), 16 originating from *B*. *rapa* (*BrCCDs)* and 14 from *B*. *oleracea* (*BoCCDs*)(S2 Table). All the genes contained sequences encoding the RPE65 domain. RPE65 belongs to a family of carotenoid oxygenases in plant, bacterial, and animal systems that typically oxidatively cleave conjugated double bonds in the polyene backbone of carotenoids [16, 17]. In addition to light related functions, the function and mechanism of RPE65 in plants have not been elucidated. To determine the evolutionary relationships among 60 CCD proteins, we constructed a phylogenetic tree by the neighbor-joining method. Based on the topology of the phylogenetic tree, CCD proteins could be grouped into nine distinct subgroups (I, II, III, IV, V, VI, VII, VIII and IX), each of which contained one of the AtCCD proteins and the corresponding subgroup members of *B*. *rapa*, *B*. *oleracea*, and *B*. *napus* (Fig 1).

The physical and chemical properties of the BnCCD proteins are summarized in Table 1. The length of BnCCD proteins ranged from 255 amino acids (aa) (BnNCED3f) to 668 aa (BnCCD7a), with a mean length of 551 aa. The predicted molecular weights varied from 28.65 kDa (BnNCED3f) to 74.79 kDa (BnCCD7a) and the theoretical isoelectric point (PI) ranged from 4.75 (BnNCED3f) to 8.83 (BnNCED3e). Out of the 30 BnCCD proteins, 27 had PI values of less than 7, and the values of the remaining three BnCCD proteins were greater than 7. Based on the hydrophilicity index of amphoteric proteins between –0.5 ~ +0.5 (a negative GRAVY value indicates hydrophilicity and a positive value indicates hydrophobicity), all BnCCD proteins are amphiphilic proteins.

**Table 1:** The genes and encoded protein features of the 30 *BnCCDs* identified in this study.

Gene name	Gene ID	Subgroup	Chromosome	Gene length (bp)	Gene position		Number of exons	Protein length (aa)	Molecular wt. (kDa)	pI	GRAVY	Subcellular location
					Start	End						
*BnCCD1a*	*BnaA09g41150D*	I	A09	4466	28783045	28787510	13	483	55.24182	6.36	−0.175	chlo: 5, cyto: 2, vacu: 2, E.R.: 2, nucl: 1, pero: 1
*BnCCD1b*	*BnaAnng12520D*	I	Ann_random	3193	13541287	13544479	13	525	59.31523	5.70	−0.256	cyto: 7, chlo: 3, nucl: 1, mito: 1, plas: 1
*BnCCD1c*	*BnaC04g20610D*	I	C04	3267	21697508	21700774	14	525	59.35633	5.94	−0.257	cyto: 10, chlo: 2, plas: 1
*BnCCD1d*	*BnaC08g33680D*	I	C08	8770	32058916	32067685	14	612	69.62388	6.95	−0.367	chlo: 9, mito: 3, nucl: 1
*BnCCD1e*	*BnaC08g33690D*	I	C08	7652	32077226	32084877	15	611	69.52093	6.41	−0.289	chlo: 14
*BnNCED2a*	*BnaA01g09090D*	II	A01	1746	4447853	4449598	1	581	64.52248	5.33	−0.224	chlo: 9.5, chlo_mito: 5.5, cyto: 1, plas: 1, vacu: 1
*BnNCED2b*	*BnaA03g58230D*	II	A03_random	1752	1600131	1601882	1	583	64.95409	5.49	−0.237	chlo: 6, cyto: 5, plas: 1.5, cysk_plas: 1.5
*BnNCED2c*	*BnaC01g10770D*	II	C01	1746	6588592	6590337	1	581	64.51950	5.31	−0.214	chlo: 9, cyto: 4
*BnNCED2d*	*BnaC07g35240D*	II	C07	1752	37711814	37713565	1	583	64.82201	5.55	−0.243	chlo: 7, cyto: 4, plas: 1.5, cysk_plas: 1.5
*BnNCED3a*	*BnaA01g29390D*	III	A01	1662	20329204	20331002	2	553	60.99077	5.28	−0.287	mito: 10, chlo: 4
*BnNCED3b*	*BnaA03g33390D*	III	A03	1191	16162114	16163388	2	396	44.24510	4.99	−0.242	cyto: 6, cysk: 6, mito: 1
*BnNCED3c*	*BnaA05g25030D*	III	A05	1794	18611263	18613467	1	597	65.80847	5.94	−0.297	mito: 8, chlo: 6
*BnNCED3d*	*BnaC01g36910D*	III	C01	1797	36059468	36061576	1	598	65.72044	5.89	−0.276	mito: 8, chlo: 6
*BnNCED3e*	*BnaC05g39180D*	III	C05	948	37757359	37758306	1	315	34.06851	8.83	−0.291	mito: 11, chlo: 3
*BnNCED3f*	*BnaC05g39190D*	III	C05	768	37758388	37759155	1	255	28.64751	4.75	−0.217	cyto: 8, cysk: 4, plas: 1
*BnNCED3g*	*BnaC05g39200D*	III	C05	1794	37769098	37770891	1	597	65.84553	6.01	−0.318	mito: 9, chlo: 5
*BnNCED3h*	*BnaCnng32080D*	III	Cnn_random	1194	30423020	30424656	2	396	44.29825	5.09	−0.219	cysk: 8, cyto: 4, mito: 1
*BnCCD4a*	*BnaA01g09900D*	IV	A01	2020	4881104	4883125	1	596	65.70485	6.33	−0.236	chlo: 12, mito: 1.5, cyto_mito: 1.5
*BnCCD4b*	*BnaA08g09110D*	IV	A08	1782	8767981	8769762	3	532	58.57082	6.16	−0.129	chlo: 12, cyto: 1
*BnCCD4c*	*BnaC01g11550D*	IV	C01	2067	7220281	7222347	1	593	65.43358	6.45	−0.234	chlo: 12, mito: 1.5, cyto_mito: 1.5
*BnNCED5a*	*BnaA09g26450D*	V	A09	1170	19605914	19607683	1	589	65.33387	5.37	−0.324	chlo: 11, nucl: 2
*BnNCED5b*	*BnaCnng38510D*	V	Cnn_random	1170	37143600	37145369	1	589	65.45993	5.42	−0.320	chlo: 11, nucl: 1, mito: 1
*BnNCED6a*	*BnaA07g06050D*	VI	A07	1758	6379220	6380977	1	585	65.00222	6.07	−0.321	chlo: 7.5, chlo_mito: 6.5, mito: 4.5
*BnNCED6b*	*BnaC07g07580D*	VI	C07	1758	11978406	11980163	1	585	65.06919	6.00	−0.329	pero: 8, chlo: 3, cyto: 2
*BnCCD7a*	*BnaA04g26000D*	VII	A04	5803	18550548	18556350	10	668	74.78965	5.77	−0.381	chlo: 9, plas: 3, mito: 1
*BnCCD7b*	*BnaC04g50070D*	VII	C04	3912	47874505	47878416	9	648	72.69485	6.19	−0.303	chlo: 8, plas: 3, mito: 1, vacu: 1
*BnCCD8a*	*BnaA01g04140D*	VIII	A01	3287	1908477	1911763	6	569	63.98902	7.13	−0.341	chlo: 10, mito: 3
*BnCCD8b*	*BnaC01g05600D*	VIII	C01	12036	2963042	2975077	7	574	64.98737	8.50	−0.452	chlo: 7, cyto: 4, nucl: 2
*BnNCED9a*	*BnaA07g39250D*	IX	A07_random	1809	2059180	2060988	1	602	66.75458	5.90	−0.299	chlo: 14
*BnNCED9b*	*BnaC06g38870D*	IX	C06	1809	36264799	36266607	1	602	66.79169	6.11	−0.281	chlo: 14

chlo: chloroplast; cyto: cytoplasmic; vacu: vacuolar; E.R: Endoplasmic Reticulum; pero: peroxisomal; mito: mitochondria; nucl: nucleus; plas: plasma membrane.

### Chromosomal localization and gene structure

**Fig 2:**
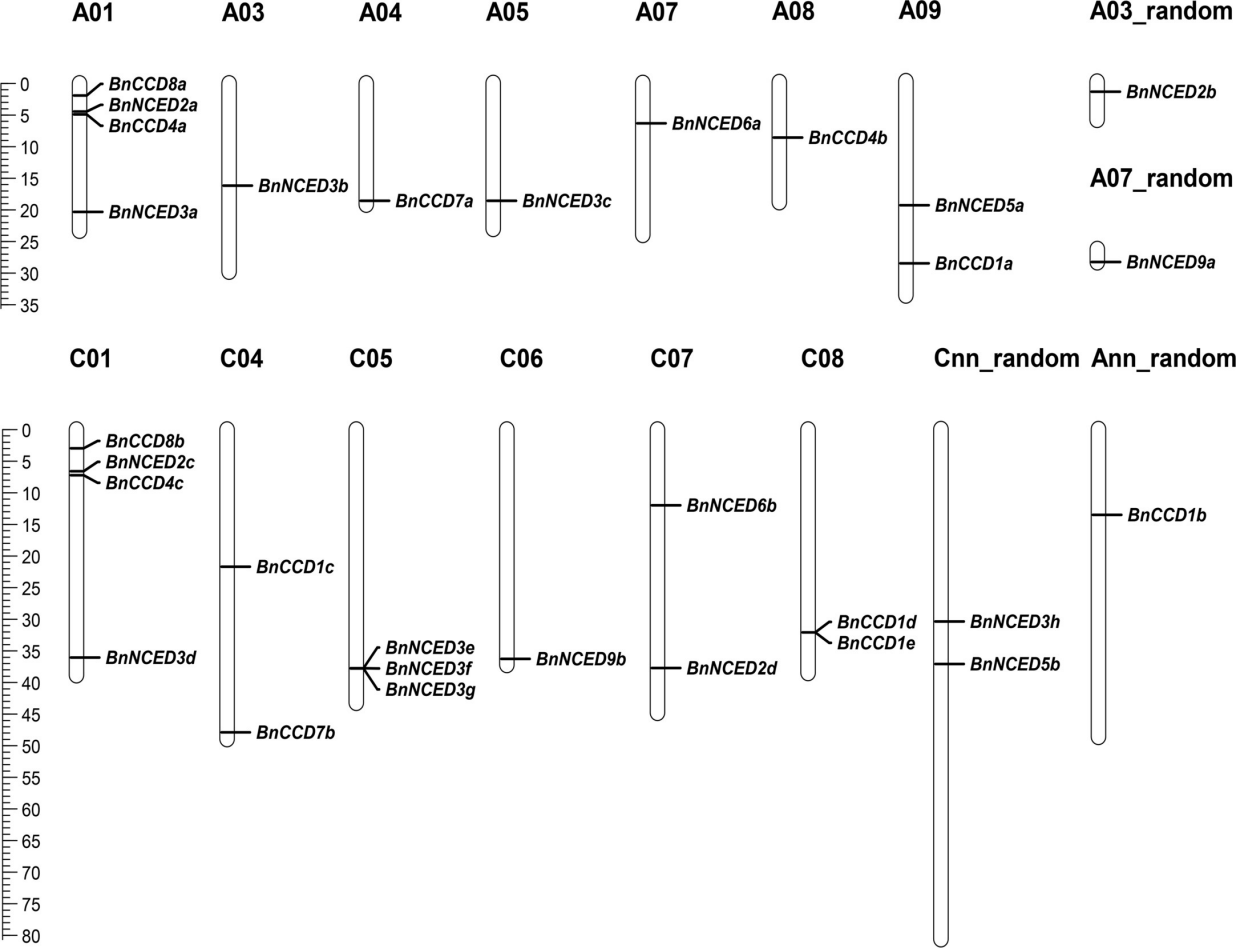
Genomic distribution of *BnCCD* genes. The chromosomal location of each *BnCCD* locus was mapped to the *B*. *napus* genome: the chromosome number is indicated above each chromosome. The scale is in megabases (Mb). Ann and Cnn are pseudo-molecule chromosomes. Random means that the specific location of the gene is unknown.

By Mapchart, an analysis of the chromosomal distribution of the *BnCCD* loci showed that these genes were not evenly distributed across the chromosomes (Fig 2). The 30 *BnCCD* loci are distributed on 13 chromosomes in *B*. *napus*; the 14 *BnCCDs* are located on the A subgenome and the 16 *BnCCDs* are located on the C subgenome. Chromosomes A01 and C01 possess the most *BnCCD* loci, each containing four *BnCCDs*. Chromosomes A03, A04, A05, A07, A08, C06, A03-random, A07-random, and Ann-random each contain only one *BnCCD* locus, whereas chromosomes A09, C04, C07, C08, and Cnn-random each contain two *BnCCD* loci. *BnNCED3e*, *BnNCED3f*, and *BnNCED3g* (subgroup III) are located on chromosome C05.

**Fig 3:**
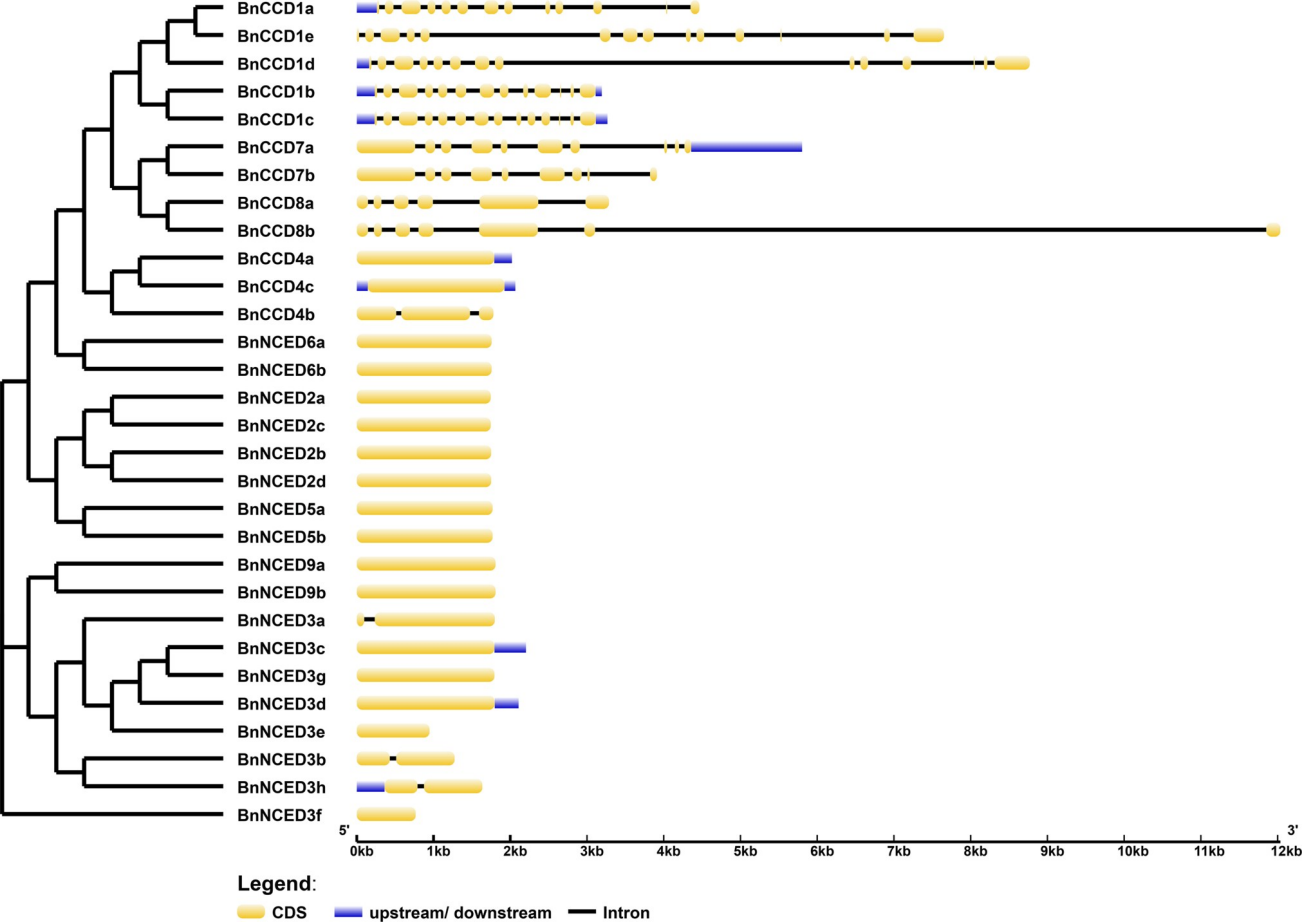
Exon–intron structures of the *BnCCD* genes, arranged based on their phylogenetic relationships. An unrooted phylogenetic tree was constructed based on the full-length sequences of BnCCD proteins using the N-J method in MEGA7. Bootstrap values based on 1,000 replications were calculated. The exon–intron structure of the *BnCCD* genes was analyzed using the GSDS online tool. The lengths of the exons and introns for each *BnCCD* gene are drawn to scale.

The positions of exons and introns in members of a gene family might have played crucial roles during evolution [62]. Therefore, we next analyzed the gene structure (exon–intron organization) of the 30 *BnCCDs* (Fig 3). Genes with similar structures were closely related. *BnNCED2*, *BnNCED5*, *BnNCED6*, and *BnNCED9* subgroups were intron-less. The *BnCCD1* subgroup contained 13–15 exons; two genes contained 13 exons, two genes contained 14 exons, and only *BnCCD1e* possessed 15 exons. All *BnCCD4* subgroup members contained one exon, except for *BnCCD4b*, which contained three. *BnCCD8b* contained 6 introns, one more than *BnCCD8a*.

### Conserved motifs and protein profiles of BnCCD proteins

**Fig 4:**
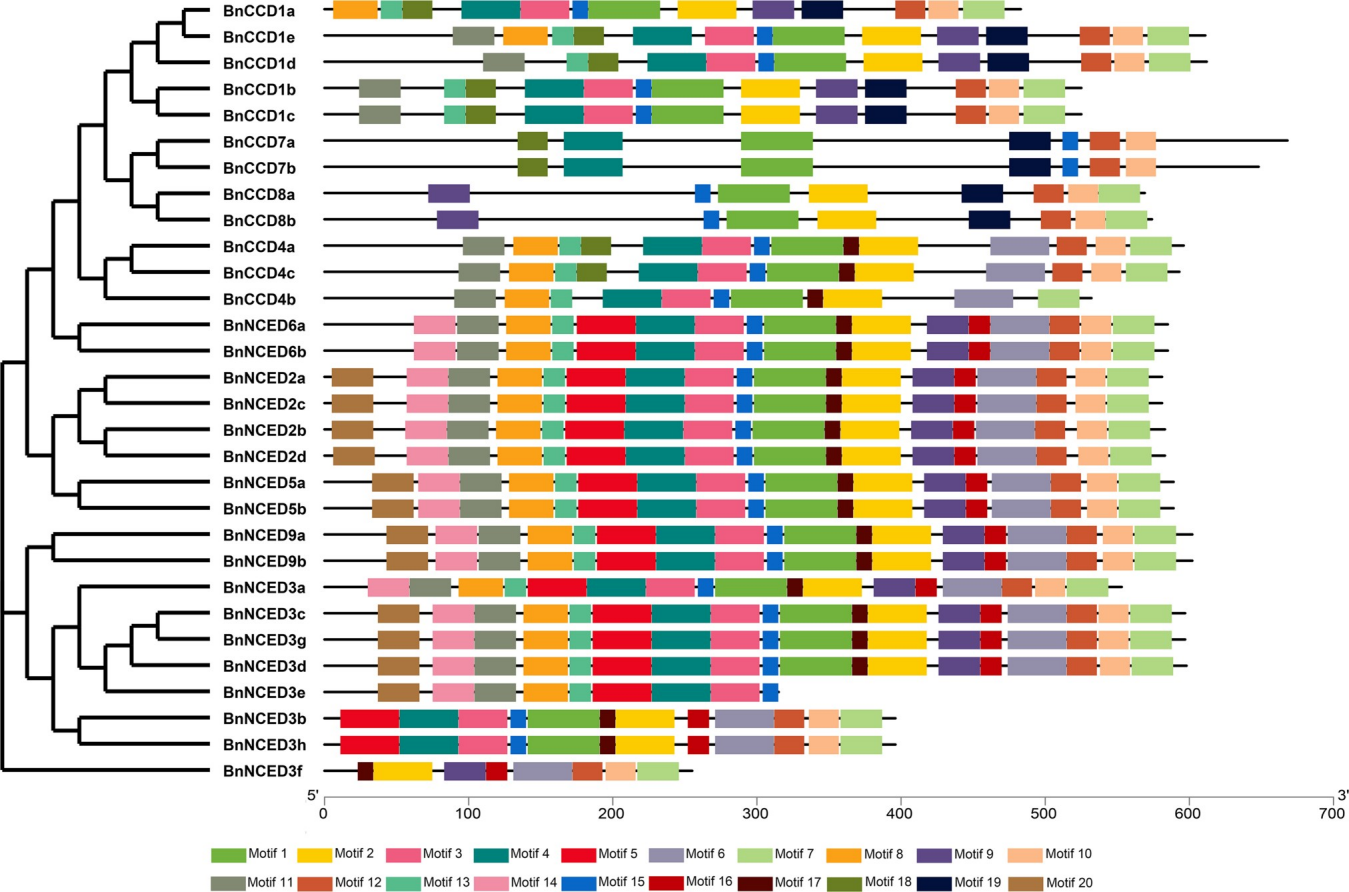
The conserved motifs of the BnCCD proteins, arranged based on their phylogenetic relationships. The conserved motifs of the BnCCD proteins were identified by MEME. Gray lines represent non-conserved sequences and each motif is indicated by a colored box (numbered at the bottom). The lengths of the motifs in each protein are drawn to scale.

Twenty putative BnCCD protein motifs were predicted by the MEME program (Fig 4). The BnNCED2, NCED5, NCED6, and NCED9 proteins all contain motifs 1–17. Except for BnNCED3e, all other BnNCED3 proteins contain motifs 2, 6, 7, 10, 12, 16, and 17. Motifs 1, 2, 7, and 15 are present in all BnCCD1, BnCCD4, and BnCCD8 proteins and all BnCCD7 members contain motifs 1, 4, 10, 12, 15, 18, and 19. Using the InterProScan program, we searched for annotations for the conserved motifs. Motifs 1–9 and 19 were associated with carotenoid oxygenase (IPR004294) and the remaining motifs had not been annotated.

Prediction of secondary structure using SOPMA showed that the main structure of the BnCCD proteins was a random coil. In addition to BnNCED3e, the alpha helix was more than the extension chain. The alpha helix was equal to the extension chain in BnCCD7a. The secondary structure characteristics of other BnCCD proteins ranged from more to less: random curl, extended strand, alpha helix, beta turn. Analysis using the TMHMM Server v. 2.0 showed that no BnCCD proteins possessed a transmembrane domain.

Nineteen of the 30 BnCCD proteins were predicted to be localized to the chloroplast, five to the cytoplasm, and five to the mitochondrion. BnNCED6b was predicted to be localized to the peroxisome.

### *Ka* and *Ks* calculation for orthologous *CCD* genes between *Brassica napus* and *Arabidopsis thaliana*

To determine whether the *CCD* protein-coding genes in *B*. *napus* and *A*. *thaliana* are under selective pressure, the *Ka/Ks* ratios for 30 pairs of orthologous genes were calculated using TBtools software (Table 2). The *Ka/Ks* ratios of all the gene pairs were considerably lower than 1, indicating that the *CCD* gene family has undergone purifying selection. The *Ks* values for *B*. *napus* relative to *A*. *thaliana* ranged from 0.28852 to 0.65433, suggesting that gene duplications occurred approximately 9.62–21.81 million years ago (MYA).

**Table 2:** The non-synonymous (*Ka*) and synonymous substitution rate (*Ks*) for orthologous *CCD* gene pairs between *B*. *napus* and *A*. *thaliana*.

Orthologous gene pairs		*Ka*	*Ks*	*Ka/Ks*	Duplication date (MYA)
*AtCCD1*	*BnCCD1a*	0.08151	0.36209	0.22510	12.07
*AtCCD1*	*BnCCD1b*	0.04122	0.32012	0.12875	10.67
*AtCCD1*	*BnCCD1c*	0.03384	0.28852	0.11730	9.62
*AtCCD1*	*BnCCD1d*	0.05913	0.38774	0.15250	12.92
*AtCCD1*	*BnCCD1e*	0.07611	0.35530	0.21422	11.84
*AtNCED2*	*BnNCED2a*	0.06843	0.46502	0.14716	15.50
*AtNCED2*	*BnNCED2b*	0.04285	0.41304	0.10374	13.77
*AtNCED2*	*BnNCED2c*	0.06108	0.47780	0.12784	15.93
*AtNCED2*	*BnNCED2d*	0.04606	0.43371	0.10620	14.46
*AtNCED3*	*BnNCED3a*	0.04945	0.56001	0.08829	18.67
*AtNCED3*	*BnNCED3b*	0.05042	0.55742	0.09045	18.58
*AtNCED3*	*BnNCED3c*	0.05052	0.51718	0.09768	17.24
*AtNCED3*	*BnNCED3d*	0.04962	0.52187	0.09509	17.40
*AtNCED3*	*BnNCED3e*	0.05472	0.45159	0.12116	15.05
*AtNCED3*	*BnNCED3f*	0.05271	0.58288	0.09043	19.43
*AtNCED3*	*BnNCED3g*	0.04581	0.51353	0.08921	17.12
*AtNCED3*	*BnNCED3h*	0.05693	0.55439	0.10268	18.48
*AtCCD4*	*BnCCD4a*	0.07819	0.59005	0.13252	19.67
*AtCCD4*	*BnCCD4b*	0.06912	0.50381	0.13720	16.79
*AtCCD4*	*BnCCD4c*	0.07461	0.60924	0.12246	20.31
*AtNCED5*	*BnNCED5a*	0.04001	0.38163	0.10484	12.72
*AtNCED5*	*BnNCED5b*	0.03847	0.40056	0.09605	13.35
*AtNCED6*	*BnNCED6a*	0.08570	0.57248	0.14970	19.08
*AtNCED6*	*BnNCED6b*	0.09078	0.57371	0.15824	19.12
*AtCCD7*	*BnCCD7a*	0.07106	0.45309	0.15683	15.10
*AtCCD7*	*BnCCD7b*	0.07166	0.42570	0.16834	14.19
*AtCCD8*	*BnCCD8a*	0.05711	0.42701	0.13374	14.23
*AtCCD8*	*BnCCD8b*	0.09405	0.49007	0.19191	16.34
*AtNCED9*	*BnNCED9a*	0.07262	0.61567	0.11796	20.52
*AtNCED9*	*BnNCED9b*	0.06799	0.65433	0.10391	21.81

### The *cis*-acting elements predicted to be present in *BnCCD* promoters

The *cis*-acting elements present in the promoters are essential for transcriptional gene regulation. In total, 87 types of *cis*-acting elements were predicted to be present in 30 *BnCCD* promoters (S3 Table), using the online software PlantCARE (http://bioinformatics.psb.ugent.be/webtools/plantcare/html/). In addition to CAAT and TATA boxes, each promoter contained more than 10 *cis*-acting elements. Among these, 26 different elements are associated with responses to light, with 206 occurrences across all promoters. The remaining elements are associated with hormonal and stress responses and tissue-specific expression. Hormone-responsive *cis*-elements account for a large proportion of the total and include ABRE (abscisic acid response element), AuxRR-core and TGA-elements (auxin response), ERE (ethylene-response element), TCA-element (salicylic acid response), P-box and GARE-motif (gibberellin-response), and the CGTCA and TGACG motifs (MeJA-response). Notably, all *BnCCDs* contain ABRE, CGTCA, and TGACG motifs, suggesting that this gene family functions in hormone response pathways. Additional *cis*-elements related to stress response were identified; for example, LTR (low-temperature responsiveness) is present in all *BnCCD* promoters except for *BnNCED3f* and *BnCCD8a*. Additional elements include the drought-inducible MBS element, TC-rich repeats, a *cis*-acting element involved in defense and stress responsiveness, and the WUN-motif, a wound-responsive element.

Some *cis*-acting elements in *BnCCD* promoters might determine the tissue-specific expression pattern, such as the CAT-box, which is associated with meristematic expression; GCN4_motif, involved in endosperm expression; HD-Zip 1, involved in differentiation of the palisade mesophyll cells; and RY-element, which confers seed-specific expression.

### RNA-seq analysis

**Fig 5:**
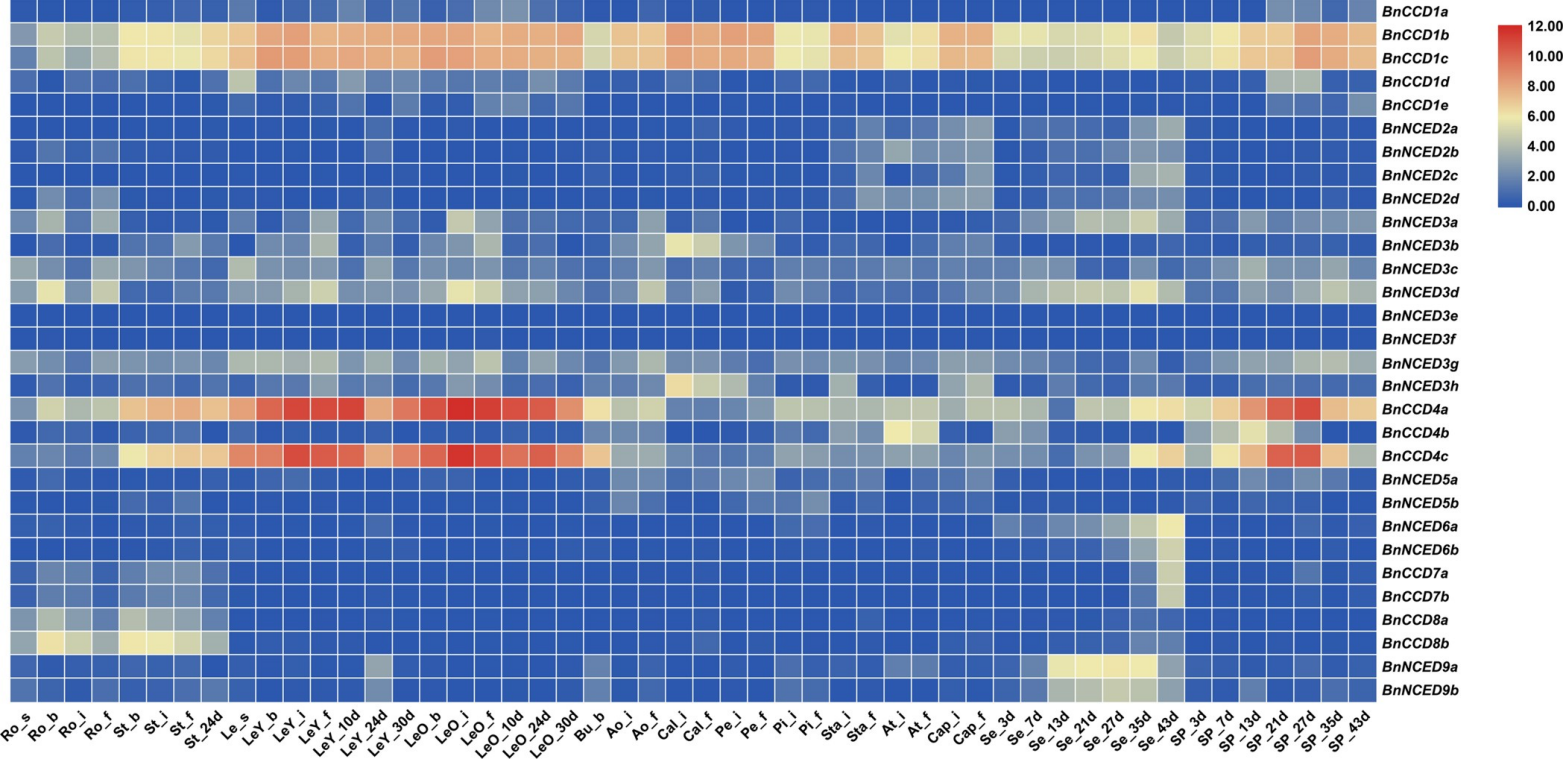
Heatmap of the expression patterns of selected *BnCCD* genes in different tissues and at different developmental stages. The expression data were obtained from publically available RNA-sequening (RNA-seq) data. Ro: Roots; St: Stems; Le: Leaves; LeY: Young leaves; LeO: Old leaves; Bu: Buds; Ao: Anthocauli; Cal: Calyxes; Pe: Petals; Pi: Pistils; Sta: Stamens; At: Anthers; Cap: Capillaments; Se: Seeds; Sp: Silique pericarps; _s: At seedling stage; _b: In the bud stage; _i: At the initial flowering stage; _f: At the flourishing flowering stage; _3d: 3 days after flowering; _7,10,13,21,24,27,30,35,43d: 7,10,13, 21,24,27,30,35,43days after flowering; Ro_s: Roots at seedling stage.

We analyzed the expression of 30 *BnCCDs* at different developmental stages and in various tissues from the *B*. *napus* cultivar ZS11 and constructed an expression heat map (Fig 5). Whereas no *BnNCED3f* and *BnNCED3g* transcripts were detected in any tissue, the remaining 28 *BnCCDs* showed tissue- and development-specific expression levels. *BnCCD1b* and *BnCCD1c* were highly expressed in leaves, stems, buds, flowers, seeds, and silique pericarp, but expression was relatively low in roots. *BnNCED9a* and *BnNCED9b* had similar expression patterns and were highly expressed in seeds. Genes belonging to the same subgroup had a wide range of expression patterns: *BnCCD4a* and *BnCCD4c* were highly expressed in leaves, stems, and pericarps; however, *BnCCD4b*, which belongs to the *BnCCD4* subgroup, had a low expression level in leaves, stems, and pericarps but was highly expressed in anthers, suggesting that it might function in stamen growth and development.

### Expression profiling of *BnCCDs* in different organs

**Fig 6:**
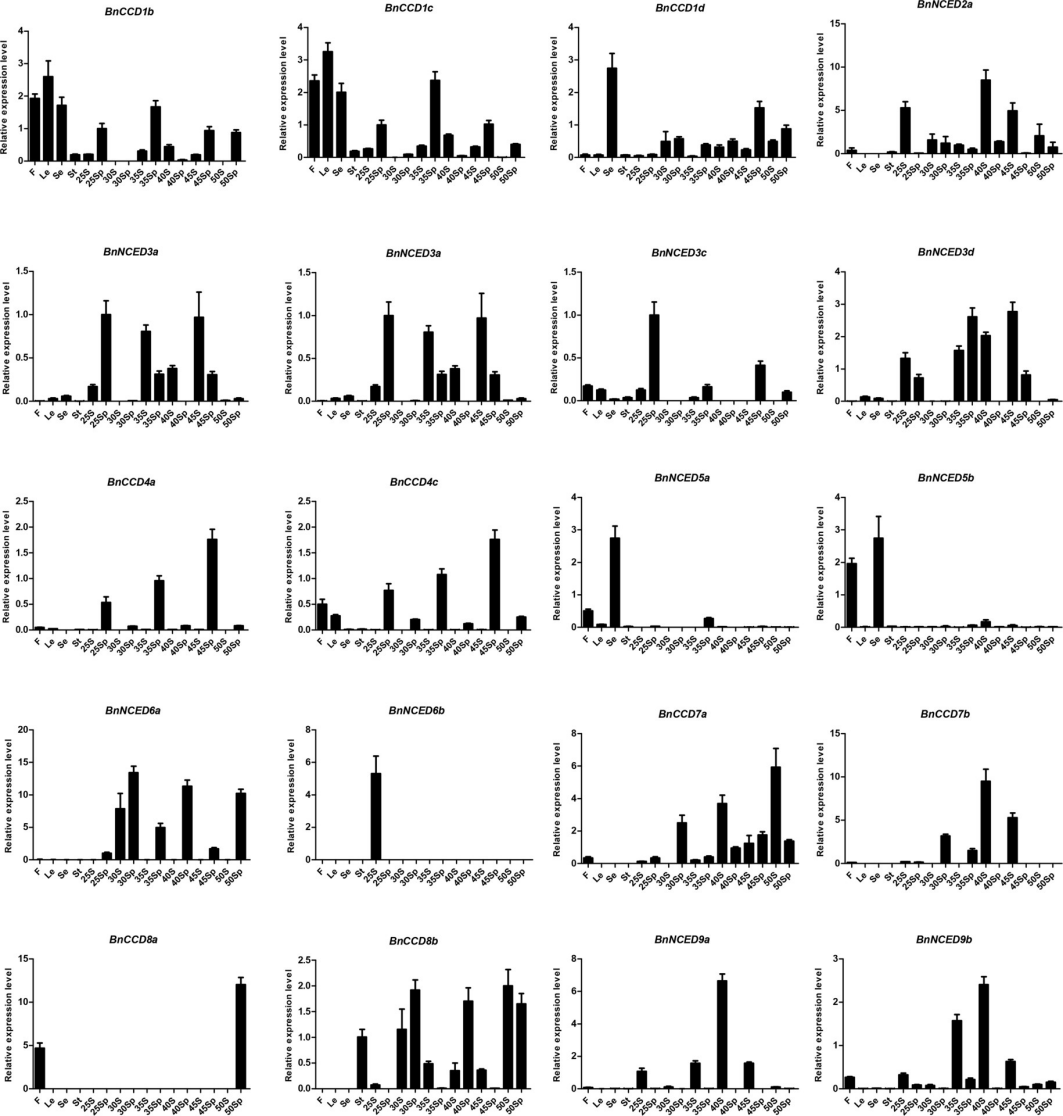
The expression patterns of selected *BnCCD* genes in different tissues and at different developmental stages. The expression patterns of 20 *BnCCD* genes in *B*.*napus* cultivar Zhong Shuang 11 (ZS11) were analyzed by qRT-PCR. F: flowers; Le: leaves; Se: sepals; St: stems; 25S, 30S, 35S, 40S, 45S, 50S: seeds collected at 25, 30, 35, 40, 45and 50 days after flowering, respectively. 25Sp, 30Sp, 35Sp, 40Sp, 45Sp, 50Sp: silique pericarps collected at 25, 30, 35, 40, 45 and 50 days after flowering, respectively. Values represented the mean ± standard error of mean (SEM) of three biological replicates with three technical replicates at each developmental stage. Error bars indicated the SEM among three experiments.

To gain insight into the biological functions of *BnCCDs*, we analyzed their expression patterns in flowers, leaves, sepals, stems, seeds, and silique pericarps, and for seeds and silique pericarps also at different developmental stages (25, 30, 35, 40, 45, and 50 days after flowering) by qRT-PCR. Several *BnCCDs*, including *BnCCD1b*, *BnCCD1c*, *BnCCD1d*, *BnNCED2a*, *BnNCED2c*, *BnNCED3a*, *BnNCED3c*, *BnNCED3d*, *BnCCD4a*, *BnCCD4c*, *BnNCED5a*, *BnNCED5b*, *BnNCED6a*, *BnNCED6b*, *BnCCD7a*, *BnCCD7b*, *BnCCD8a*, *BnCCD8b*, *BnNCED9a*, and *BnNCED9b*, had diverse expression patterns in different tissues (Fig 6). Except for *BnCCD8b*, the expression level of *CCD* genes was low in the stem. *BnNCED5b* and *BnCCD8a* transcripts were abundant in flowers, *BnNCED5a* and *BnNCED5b* transcripts were abundant in sepals, and *BnNCED9a* and *BnNCED9b* transcripts were abundant in seeds. However, the expression patterns of some genes within the same family differed: the transcript levels of *BnCCD1b* and *BnCCD1c* in leaves were higher than those in other tissues, whereas *BnCCD1d* was most highly expressed in sepals. In addition, *BnNCED2a* and *BnNCED2c* transcript levels were higher in seeds at 40–50 days after flowering (40S, 45S, and 50S) than at 25–35 days after flowering (25S, 30S and 35S). *BnCCD4a* and *BnCCD4c* transcript levels were abundant in silique pericarps collected at the 25 days after flowering (25Sp), 35Sp, and 45Sp stages, but were present at low levels at the 30Sp, 40Sp, 45Sp and 50Sp stages.

## Discussion

Arabidopsis contains nine *CCD* genes. Brassicaceae, including Arabidopsis, underwent an ancient α, β and γ whole-genome duplication polyploidization events, and also experienced an additional recent genome-wide tripling (WGT) event [63]. Because it is an allotetraploid formed by interspecific hybridization between *B*. *rapa* and *B*. *oleracea*, *B*. *napus* would be expected to contain six copies of each *Arabidopsis thaliana* gene (i.e., 54) [64]. Because of genome shrinkage and gene loss, the number of *BnCCDs* identified in this study was a little lower (i.e., 30), with only 2–6 copies of each Arabidopsis gene present. The *B*. *rapa* and *B*. *oleracea* genomes contain 16 *BrCCD* and 14 *BoCCD* genes, respectively, and the number of *BnCCDs* is the sum of these. However, only two members of the *BnNCED9* subgroup and three of the *BnCCD4* subgroup were identified in this study, whereas two members were found in the corresponding subgroups of both *B*. *rapa* and *B*. *oleracea*, indicating that gene loss might have occurred among subgroups *BnCCD4* and *BnNCED9*.

The molecular characteristics of all BnCCD protein members were found to differ, but proteins in the same subgroup had similar molecular weights and isoelectric points. The position of exon–intron boundaries reflects the evolution of these genes [62]. Subcellular localization predictions suggested that BnCCD proteins are localized to the chloroplast, mitochondrion, cytoplasm, and peroxisome. BnCCD1b, BnCCD1c, BnNCED3b, BnNCED3f, and BnNCED3h were predicted to be cytoplasmic, suggesting that BnCCD1 and BnNCED3 might interact in the cytoplasm. It also showed that these genes may not participate in chlorophyll photosynthesis, which was consistent with the research by Zhang *et al*. [28]. *BnNCED2* subgroup is located in the chloroplast. Studies have shown that histidine residues in NCED amino acids can bind Fe ^2+^ to make NCED proteins function [65], and there is a chloroplast transit peptide structure in them [66]. Wang *et al*. [67] found that NCED2 protein in *Camellia sinensis* is localized in chroloplast and has N-terminal chloroplast targeting signal peptide sequences, which further proves that *CsNCED2* has the biological activity of cleaving epoxy carotenoids to generate ABA precursors in plastids. Besides, in both sugarcane and rice, CCD8 are proteins located in chloroplast [25, 68]. Through online analysis we found that *BnCCD8* is also localized in the chloroplast.

Seven *cis*-acting elements associated with various stress responses were predicted among the promoters of the 30 *BnCCDs*; LTR, MBS, TC-rich repeats, and nine *cis*-acting elements were associated with responses to abscisic acid, MeJA, salicylic acid, and auxin. *CCD1*, *4*, and *8* and *NCED2* and *9* are transcriptionally upregulated following treatment of *B*. *rapa* with the phytohormones ABA and SL [16]. Furthermore, *Nicotiana tabacum* plants that heterologously expressed *NCED1* of *Stylosanthes guianensis* had increased tolerance to light, oxidative, drought, salt, and cold stress, indicating that *CCD* might regulate plant tolerance against these abiotic stresses [69]. The G-Box, GT1, and ACE motifs, which are transcriptionally responsive to light, are present in the *BnCCD* gene promoters. In addition, *BnCCD* promoters contain circadian response elements, RY-elements, and MSA-like and MBSI elements, indicating that most *CCD* genes function in plant growth and development and stress responses.

Based on RNA-seq data (S4 Table), we constructed an expression heatmap, which demonstrated that the 30 *BnCCDs* were differentially expressed in different *B*. *napus* tissues. Members of the same gene subgroup occasionally exhibited different expression patterns, such as *BnCCD1* and *BnCCD4*, potentially reflecting new functionalization among the gene family during evolution. Genes of the *CCD1* and *CCD4* subgroups function in the formation of a variety of apo-carotenoids, which confer unique colors, tastes, and aromas [70–72]. A *CCD1* loss-of-function mutant showed a decreased level of β-ionone in tomato fruit (*Solanum lycopersicum*) [31] and petunia flowers (*Petunia hybrida*) [73]. Carotenoid homeostasis is regulated by *CCD4* in different tissues, such as *Arabidopsis* seeds [74] and potato tubers [23]. *BnCCD4b* was mainly expressed in stamens, suggesting that it might function in stamen growth and development. Species such as *Solanumly copersicum*, *Prunus persica*, and *Crocus*, were also reported to preferentially express *CCD4* in floral organs, indicating that the evolution of *CCD4* genes might have been adaptive and have enhanced specific physiological traits unique to flowering plants [21, 75, 76]. *CCD4* with normal function or lack of function will change the color of fruit and flower organs. Previous studies have suggested *CCD4* gene can fade the yellow petals of *Rhododendron japonicum* and *Eustoma grandiflorum* [77, 78], because its ability of cleaving carotenoids. Inactivation of *CCD4* will change the color of *B*.*napus* and *Chrysanthemum morifolium* petals from white to yellow [79, 80]. Controlling the expression of *CCD* gene family provides a way to change the color of plant petals.

## Conclusion

In conclusion, we performed a comprehensive study of *CCD* gene family in *B*.*napus*. We identified 30 putative *BnCCDs* that were classed into nine subgroups (I-IX) on the basis of their phylogenetic relationships. The length of the BnCCD proteins ranged from 255 to 668 aa, and the Ks values for *B*. *napus* relative to *A*. *thaliana* ranged from 0.28852 to 0.65433. In addition, RNA-seq data and qRT-PCR analysis revealed that *BnCCDs* were differentially expressed in different tissues and organs, and had tissue/organ specificity and expression preference, suggesting that *BnCCDs* had clear function differentiation. Our results will help lay the foundation for the functional characterization of the *CCD* gene family and better understand the structural and functional relationships among these family members.

## Supporting information

S1 Table qRT-PCR primers used to analyze *BnCCDs* expression. (XLSX)

S2 Table List of identified *CCD* genes in *B*.*napus*, *B*.*rapa*, *B*.*oleracea and A*. *thaliana*. (XLSX)

S3 Table *Cis*-acting elements in the promoter regions of *BnCCDs*. (XLSX)

S4 Table RNA-seq data of 30 BnCCDs. (XLSX)
